# The anti-inflammation, anti-oxidative and anti-fibrosis properties of swertiamarin in cigarette smoke exposure-induced prostate dysfunction in rats

**DOI:** 10.18632/aging.102467

**Published:** 2019-11-17

**Authors:** Jinglou Chen, Jianhua Liu, Yongfang Lei, Min Liu

**Affiliations:** 1 The Gerontology Research Center of Jianghan University, The Sixth Hospital of Wuhan (Affiliated Hospital of Jianghan University), Jianghan University, Wuhan, China; 2 Medical College, Jianghan University, Wuhan, China; 3 Department of Pharmacy, Tongji Hospital, Tongji Medical College, Huazhong University of Science and Technology, Wuhan, China

**Keywords:** cigarette smoke, prostate, swertiamarin, fibrosis

## Abstract

Chronic cigarette smoke (CS) exposure induces prostate deficits. We previously found that swertiamarin had prostatic protective potential. This study was to investigate the possible protective effect of swertiamarin against CS-induced prostate dysfunction on human prostate epithelial cells, stromal cells and rats. Rat prostate collagen deposition and fibrosis were assessed by sirius red staining and measuring hydroxyproline content, as well as by qPCR and western blot analysis for fibrotic extracellular matrix components. Prostatic levels of oxidative stress and inflammatory-related factors were also analyzed. In order to explore its underling mechanisms, the activities of Hedgehog signaling pathway and epithelial-mesenchymal transition of human prostate cells and rat prostate tissue were estimated. It was found that swertiamarin ameliorated CS-induced prostatic collagen deposition, relieved oxidative stress and local inflammation, inhibited the activation of Hedgehog signaling pathway and attenuated epithelial-mesenchymal transition. It indicated that swertiamarin could ameliorate CS-induced prostatic fibrosis by inhibiting epithelial-mesenchymal transition and Hedgehog pathway.

## INTRODUCTION

Tobacco is produced from economic crop *Nicotiana tabacum* and popularly consumed (about 1.27 billion smokers) over the world [1, 2]. Cigarette smoke (CS) is suggested to have more than 4700 chemical compounds including about 60 known carcinogens. Among them, about 92% are gases (such as carbon-monoxide, nitrogen oxide, hydrogen cyanide, ozone and formaldehyde) and about 8% are particles (including naphthalene and heavy metals (like cadmium)) [3]. The volatility of cadmium helps it transfers to the CS and then be absorbed into the human body. Furthermore, cadmium has a long biological half-life and is easily accumulated *in vivo* and contributes to prostatic deficits [4, 5]. It is not only that CS exposure stimulates prostate oxidative damage, but also is a potential carcinogenic factor of prostate because it stimulates angiogenesis and promotes prostate cancer cells proliferation [6, 7].

Qing Ye Dan (QYD) is the whole plant of *Swertia mileensis* and used in Chinese folk medicine for the treatment of prostatitis, benign prostatic hyperplasia (BPH) and so on. Swertiamarin is one of the mainly bioactive substances in QYD [8]. Our previous studies confirmed that QYD and its main active ingredient swertiamarin could protect against BPH and cadmium-induced prostatic deficits due to their properties of anti-hyperplasia, anti-oxidative and anti-inflammatory [9, 10].

This study was undertaken to investigate the potential protective effects against CS-induced prostate damages and its underling mechanisms of swertiamarin on human prostate epithelial cells (RWPE-1) and human prostate stromal cells (WPMY-1), as well as on rats.

## RESULTS

### Swertiamarin ameliorated CS-induced prostatic collagen deposition

**Figure 1:**
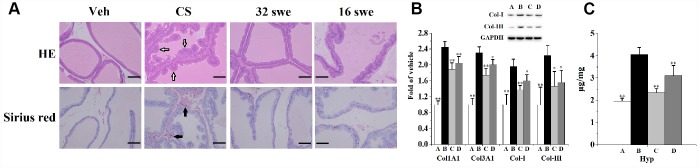
(**A**) HE and sirius red staining (n=6 per group, magnification ×200, scale bar=100 μm) for the evaluation of prostate morphological changes and collagen deposition. Hollow arrow: irregular bulges. Solid arrow: collagen deposition. (**B**) Prostatic mRNA levels (n=4 per group) of Col1A1 and Col3A1, as well as the expression (n=3 per group) of Col-I and Col-III. (**C**) Prostatic content of Hyp (n=6 per group). ^**^ *p*<0.01, ^*^ *p*<0.05 compared to the CS group. A: vehicle, B: CS, C: CS-32 mg/kg swertiamarin, D: CS-16 mg/kg swertiamarin.

It can be seen from Figure 1A that exposed to CS for 90 days leads to prostate histomorphological changes. When compared to the vehicle control, a large number of irregular bulges (hollow arrow) appeared in rats prostate from CS group. Furthermore, sirius red staining showed that CS exposure provoked prostate collagen deposition (solid arrow) compared to the vehicle control.

Hydroxyproline (Hyp) is the characteristic component of collagen tissue. When compared to the vehicle control, CS significantly enhanced (Figure 1C) the prostatic level of Hyp. Additionally, chronic CS exposure significantly increased (Figure 1B) the mRNA levels of Col1A1 and Col3A1, as well as obviously promoted the expression of collagen (Col)-I and Col-III when compared to the vehicle control.

When compared to the CS group, swertiamarin (32 and 16 mg/kg/d) ameliorated prostate morphological changes, significantly reduced the Hyp content, decreased the mRNA levels of Col1A1 and Col3A1, inhibited the expression of Col-I and Col-III. Furthermore, orally treated with 32 mg/kg/d sweriamarin (without CS exposure) for 90 days did not increase or decrease (*p*>0.05) the prostate weight, body weight, Hyp content and prostatic expression of Col-I and Col-III compared to the vehicle control (Supplementary Figure 1). In cellular experiments, 30 μmol/L sweriamarin (without CS exposure) did not increase or decrease (*p*>0.05) the expression of SHH and IHH compared to the vehicle control in RWPE-1 and WPMY-1 cells (Supplementary Figure 2). It showed that the sweriamarin (32 mg/kg/d) itself had little influence on normal prostate.

### Swertiamarin relieved CS-induced prostate oxidative stress and local inflammation

**Figure 2:**
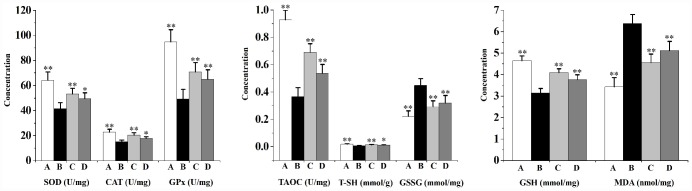
**Prostatic overall antioxidant status (TAOC and T-SH), activities of antioxidant enzymes (SOD, CAT and GPx), level of non-enzymatic antioxidant (GSH) as well as contents of oxidative stress indicators (MDA and GSSG) in rats (n=6 per group).** ^**^ *p*<0.01, ^*^ *p*<0.05 compared to the CS group. A: vehicle, B: CS, C: CS-32 mg/kg swertiamarin, D: CS-16 mg/kg swertiamarin.

As indicated in Figure 2, chronic CS exposure significantly decreases prostatic levels of the overall antioxidant status (total antioxidant capacity (TAOC) and total sulfhydryl (T-SH)), diminishes the activities of antioxidant enzymes (superoxide dismutase (SOD), catalase (CAT) and glutathione peroxidase (GPx)), reduces the level of non-enzymatic antioxidant (reduced glutathione (GSH)), as well as increases the contents of oxidative stress indicators (malondialdehyde (MDA) and oxidized glutathione (GSSG)) when compared to the vehicle control. When compared to the CS group, swertiamarin (32 and 16 mg/kg/d) significantly enhanced the activities of SOD, CAT and GPx, increased the levels of TAOC, T-SH and GSH, decreased the contents of MDA and GSSG.

**Figure 3:**
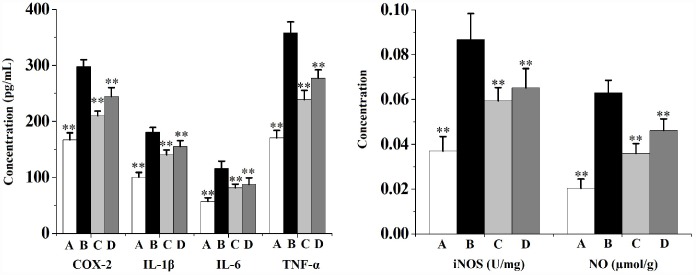
**Prostate levels of proinflammatory cytokines (IL-1β, IL-6 and TNF-α) and inflammatory-related factors (COX-2, iNOS and NO) in rats (n=6 per group).** ^**^ *p*<0.01 compared to the CS group. A: vehicle, B: CS, C: CS-32 mg/kg swertiamarin, D: CS-16 mg/kg swertiamarin.

Figure 3 shows that CS exposure results in prostate local inflammation. When compared to the vehicle control, the prostatic levels of proinflammatory cytokines (interleukin (IL)-1β, IL-6 and tumor necrosis factor (TNF)-α), as well as the levels of inflammatory-related factors (cyclooxygenase (COX)-2, inducible nitric oxide synthase (iNOS) and NO) in rats from CS group were significantly increased. Swertiamarin (32 and 16 mg/kg/d) significantly decreased the levels of IL-1β, IL-6, TNF-α and NO, as well as inhibited the activities of COX-2 and iNOS compared to the CS group.

### Swertiamarin inhibited prostatic epithelial-mesenchymal transition (EMT) and Hedgehog (HH) signaling pathway in rats

**Figure 4:**
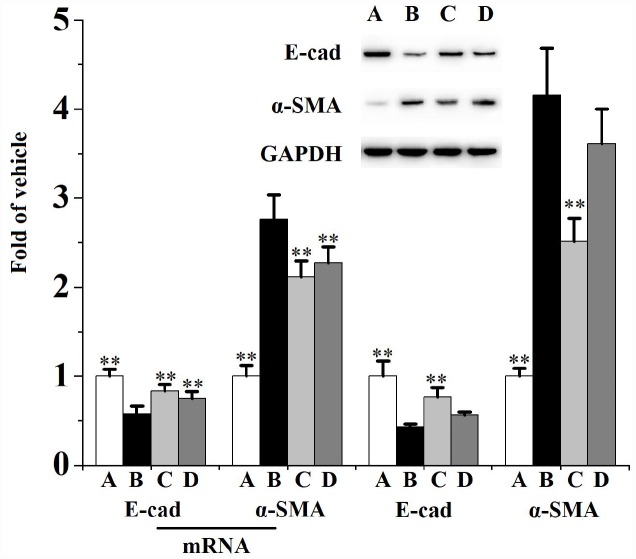
**Prostatic mRNA levels (n=4 per group) and expression (n=3 per group) of E-cad and α-SMA in rats.** ^**^p<0.01 compared to the CS group. A: vehicle, B: CS, C: CS-32 mg/kg swertiamarin, D: CS-16 mg/kg swertiamarin.

**Figure 5:**
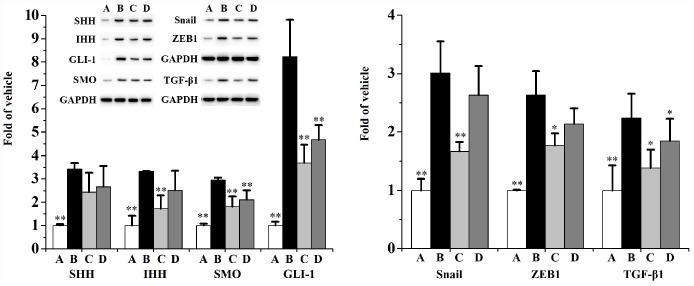
**Prostatic expression (n=3 per group) of SHH, IHH, SMO, GLI-1, Snail, ZEB1 and TGF-β1.** ^**^ *p*<0.01, ^*^ *p*<0.05 compared to the CS group. A: vehicle, B: CS, C: CS-32 mg/kg swertiamarin, D: CS-16 mg/kg swertiamarin.

Figures 4 and 5 describes the inhibiting functions of swertiamarin on prostatic EMT and HH signaling pathway. When compared to the vehicle control, exposed to CS for 90 days significantly (Figure 4) enhanced the mRNA level and protein expression of α-smooth muscle actin (α-SMA), reduced the mRNA level and protein expression of E-cadherin (E-cad). Additionally, chronic CS exposure significantly (Figure 5) activated the prostatic expression of Sonic HH (SHH), Indian HH (IHH), Smoothened (SMO), Glioma-associated oncogene homolog (GLI)-1, Snail, ZEB1 and transforming growth factor (TGF)-β1 compared to the vehicle control.

When compared to the CS group, treated with 32 mg/kg/d swertiamarin for 90 days significantly inhibited the prostatic expression of α-SMA, IHH, SMO, GLI-1, Snail, ZEB1 and TGF-β1, as well as enhanced the expression of E-cad. Both 32 and 16 mg/kg/d swertiamarin decreased the mRNA level of α-SMA and increased the mRNA level of E-cad compared to the CS group.

### Swertiamarin inhibited CS-induced human prostate cells proliferation

**Figure 6:**
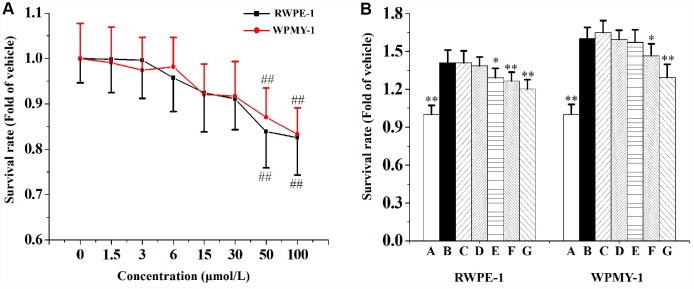
(**A**) The effects of 1.5, 3, 6, 15, 30, 50 or 100 μmol/L swertiamarin (swe) on RWPE-1 and WPMY-1 cells survival rate (n=5 per group). (**B**) The functions of 1.5, 3, 6, 15 or 30 μmol/L swe on 5% CS-induced RWPE-1 and WPMY-1 cells proliferation (n=5 per group). ^##^ *p*<0.01 compared to the vehicle. ^**^ *p*<0.01, ^*^ *p*<0.05 compared to 5% CS. A: vehicle, B: 5% CS, C: 5% CS-1.5 μmol/L swe, D: 5% CS-3 μmol/L swe, E: 5% CS-6 μmol/L swe, F: 5% CS-15 μmol/L swe, G: 5% CS-30 μmol/L swe.

Figure 6A represents the effects of swertiamarin on RWPE-1 and WPMY-1 cells proliferation. It was found that cultured with 1.5, 3, 6, 15 or 30 μmol/L swertiamarin for 48 h did not increase or decrease (*P*>0.05) the survival rates of RWPE-1 and WPMY-1 cells compared to the vehicle control. However, cultured with 50 or 100 μmol/L swertiamarin for 48 h significantly decreased the survival rates of RWPE-1 and WPMY-1 cells compared to the vehicle control.

When compared to the vehicle control, 5% CS significantly promoted the growth of RWPE-1 and WPMY-1 cells (Figure 6B). Co-incubated with 6, 15 or 30 μmol/L swertiamarin for 48 h significantly inhibited CS-induced RWPE-1 cells proliferation. And co-incubated with 15 or 30 μmol/L swertiamarin significantly inhibited CS-induced WPMY-1 cells proliferation.

**Figure 7:**
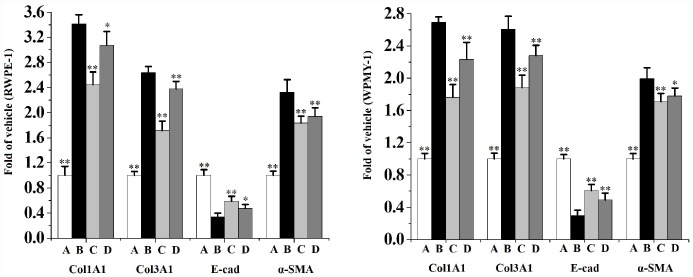
**The mRNA levels of Col1A1, Col3A1, E-cad and α-SMA in RWPE-1 and WPMY-1 cells (n=4 per group).** ^**^ *p*<0.01, ^*^ *p*<0.05 compared to 5% CS. A: vehicle, B: 5% CS, C: CS-30 μmol/L swertiamarin, D: CS-15 μmol/L swertiamarin.

As can be seen from Figure 7, co-incubated with 5% CS significantly increases the mRNA levels of α-SMA, Col1A1 and Col3A1, as well as decreases the mRNA level of E-cad in RWPE-1 and WPMY-1 cells compared to the vehicle control. When compared to the CS group, swertiamarin (15 and 30 μmol/L) significantly decreased the mRNA levels of α-SMA, Col1A1 and Col3A1, as well as increased the mRNA level of E-cad in 5% CS-exposed RWPE-1 and WPMY-1 cells.

**Figure 8:**
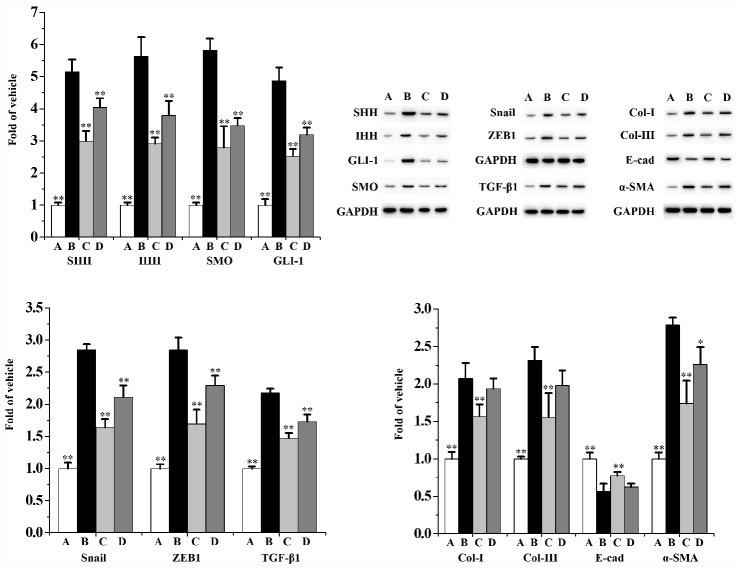
**The expression of SHH, IHH, SMO, GLI-1, Snail, ZEB1, TGF-β1, Col-I, Col-III, E-cad and α-SMA in RWPE-1 cells (n=3 per group).** ^**^ *p*<0.01, ^*^ *p*<0.05 compared to 5% CS. A: vehicle, B: 5% CS, C: CS-30 μmol/L swertiamarin, D: CS-15 μmol/L swertiamarin.

**Figure 9:**
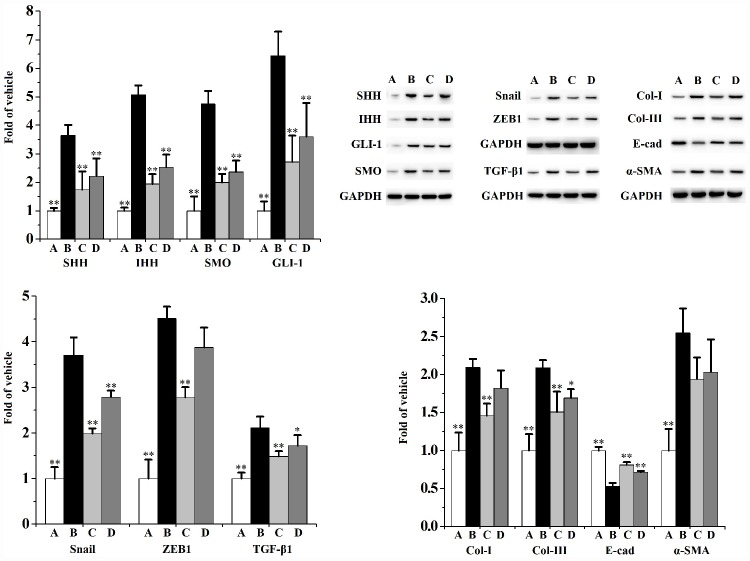
**The expression of SHH, IHH, SMO, GLI-1, Snail, ZEB1, TGF-β1, Col-I, Col-III, E-cad and α-SMA in WPMY-1 cells (n=3 per group).** ^**^ *p*<0.01, ^*^ *p*<0.05 compared to 5% CS. A: vehicle, B: 5% CS, C: CS-30 μmol/L swertiamarin, D: CS-15 μmol/L swertiamarin.

Figures 8 and 9 indicates that CS significantly activates the expression of SHH, IHH, SMO, GLI-1, ZEB1, TGF-β1, Snail, Col-I, Col-III and α-SMA, as well as diminishes the expression of E-cad in RWPE-1 (Figure 8) and WPMY-1 (Figure 9) cells compared to the vehicle control. When compared to the CS group, swertiamarin (30 μmol/L) significantly decreased the expression of SHH, IHH, SMO, GLI-1, ZEB1, TGF-β1, Snail, Col-I and Col-III, as well as increased the expression of E-cad in 5% CS-exposed RWPE-1 and WPMY-1 cells. Furthermore, swertiamarin (15 and 30 μmol/L) significantly decreased the expression of α-SMA in 5% CS-exposed RWPE-1 and WPMY-1 cells. Additionally, the specific SMO inhibitor GDC-0449 could mimic the effect. As indicated in Supplementary Figure 3, when compared to the CS model group, GDC-0449 (10 μmol/L) significantly decreased the expression of SMO and TGF-β1 in RWPE-1 and WPMY-1 cells.

## DISCUSSION

LUTS, characterized by a number of bladder and/or voiding dysfunctions (including increased nocturnal urination, frequent daytime voiding, urination weak and not finishing urination), are common disorders in elderly men [11]. LUTS severely restrict the patients quality of life and bring great economic burden [12]. It is known that the prostate is an important organ of male genitourinary system. Prostate disorders (benign or malignant) are considered to be major risk factors for benign prostatic obstruction and LUTS [11]. Fibrosis is an aberrant wound healing process. It leads to the deposition of excess extracellular matrix (ECM) [12]. Prostate become less pliable due to tissue ECM remodeling and collagen deposition. Thus, prostate fibrosis has been identified as a contributing factor for LUTS [11, 13]. EMT is the biological process of cell transformation from epithelial phenotype to mesenchymal phenotype. It is accompanied with decreased generation of E-cad and over-production of mesenchymal marker α-SMA [14]. In prostate fibrosis, myofibroblasts stimulate the expression of α-SMA and Col-I when suffered from epithelial injury [15]. In our present study, swertiamarin ameliorated chronic CS exposure-induced prostatic morphological changes, relieved collagen deposition, reduced the mRNA levels and the expression of ECM collagens, decreased the level of ECM characteristic component Hyp, as well as attenuated EMT. It indicated that swertiamarin had potential protective effect against CS-caused LUTS.

CS contains a huge number of chemicals that produce reactive oxygen species (ROS). The resulted oxidative stress is responsible for most of its damages [6]. The redox homeostasis *in vivo* is maintained by the physiological balance of ROS production and elimination. However, this balance is broken when ROS over-produced and endogenous antioxidant mechanisms diminished. Finally, oxidative stress emerges and causes pathological changes in intracellular substances such as proteins, lipids, and DNA [16]. Superoxide anions are enzymatic or non-enzymatic converted to hydroxyl radical, peroxyl radical, hydrogen peroxide and so on. For example, SOD prevents the formation of hydroxyl radical, which is highly reactive with lipids. CAT is found in peroxisomes and catalyzes the conversion of hydrogen peroxide to oxygen and water. GPX helps to inhibit lipid peroxidation. GSH, a low-molecular weight tripeptide, is the prime non-enzymatic antioxidant in the reproductive system. GSH protects against the peroxidation of lipid membrane by conjugating with the electrophile [6, 17]. MDA is the product of lipid peroxidation induced by ROS [18]. Studies found that the prostate level of MDA was remarkable elevated and the level of GSH is depleted after CS exposure [6, 19].

Altered redox homeostasis is the promoting factor for inflammation [17]. Prostatic oxidative stress contributes to the appearance and maintenance of inflammation, and ultimately promotes the pathophysiology of prostate diseases [16]. Chronic inflammation disrupts the balance of cell proliferation and apoptosis, stimulates proliferation and angiogenesis [20]. Proinflammatory cytokine TNF-α is a potent growth factor for prostatic epithelial and stromal cells [21]. ROS trigger inflammatory process *via* activating COX-2 and iNOS, as well as promoting the synthesis of NO, TNF-α and IL-1β. Prostatic inflammation exacerbates the formation of ROS in turn [14, 20]. Unfortunately, smoking stimulates both oxidative stress and inflammation in prostate [18]. We found that swertiamarin enhanced the prostate overall antioxidant status, increased the activities of antioxidant enzymes and the level of non-enzymatic antioxidant, decreased the contents of oxidative stress indicators, as well as reduced the levels of proinflammatory cytokines and inflammatory-related factors in CS-exposed rats. It seems that the antioxidant and anti-inflammatory properties of swertiamarin contribute to its prostate protective functions against CS exposure.

CS creates a vicious circle with a mutual promotion of oxidative stress and inflammation. They are the causes of ECM metabolism disorder and fibrosis [16]. Especially for prostate, local inflammation promotes collagen deposition and fibrosis, finally contributes to LUTS [12, 13]. In the network crosslinking of inflammation, oxidative stress and fibrosis, HH signaling pathway seems to stand at the node. HH signaling pathway participates in regulating a series of cell activities, biological processes and tissue homeostasis. It has been observed that HH signaling is activated in CS-induced airway inflammation [22]. HH pathway activates EMT, collagen deposition and fibrosis through triggering the transcription of its target genes Snail, Col-I and TGF-β1 [23]. In this study, swertiamarin lessened the expression of HH signaling indicators and its major target genes activated by CS.

In conclusion, swertiamarin ameliorated CS-induced prostatic collagen deposition, relieved oxidative stress and local inflammation, inhibited the activation of HH pathway, as well as attenuated EMT. It provided new insights for understanding the CS-induced prostatic deficits and added evidences for the prostatic protective nature of swertiamarin.

## MATERIALS AND METHODS

### Reagents

Cigarettes (Hongjinlong brand, the content of nicotine, tar and carbon monoxide was 0.6 mg, 8 mg and 11 mg/cigarette, respectively) were obtained from China Tobacco Hubei Industrial LLC. (Hubei, China). Swertiamarin (purity>98%) was purchased from Aladdin Co, Ltd. (Shanghai, China). RWPE-1 and WPMY-1 were obtained from China Center for Type Culture Collection (Shanghai, China). The antibodies of SHH (ab19897), IHH (ab52919), GLI-1 (ab49314), SMO (ab113438), α-SMA (ab5694), E-cad (ab1416), Col-I (ab34710), Col-III (ab7778), Snail (ab180714) and ZEB1 (ab81972) were obtained from Abcam, Co. (UK). The antibody of TGF-β1 (21898-1-AP) was purchased form PTG, Co. (Wuhan, China). ELISA kits for the analysis of COX-2 (JYM0885Ra), IL-1β (JYM0419Ra), IL-6 (JYM0646Ra) and TNF-α (JYM0635Ra) were purchased from ELISA Lab, Co. Ltd. (Hubei, China). The commercial kits for the analysis of iNOS (A014-1-2), NO (A012-1-2), TAOC (A015-2-1), T-SH (A063-1-1), MDA (A003-1-2), SOD (A001-1-2), CAT (A007-1-1), GPx (A005-1-2), GSH/GSSG (A061-1-2) and Hyp (A030-2-1) were purchased from Nanjing Jiancheng Bioengineering Institute (Nanjing, Jiangsu, China).

### Rat model of CS exposure

Male Wistar rats with the body weight of 160~180 g were purchased from Liaoning Changsheng Biotechnology Co., Ltd and fed in SPF barrier environment (Laboratory Animal Center of Huazhong Agricultural University, Permit NO. 00268872). All the experiments were performed in accordance with the Chinese legislation and the ethical rules of NIH Guidelines for the Care and Use of Laboratory Animal and were approved by the ethics committee of the Affiliated Hospital of Jianghan University. Animals were maintained at the temperature of 22±3°C and humidity of 50±10% with a 12 h light:12 h dark cycle for 1 week prior to be used in experiments. Then, the animals were randomly assigned into four groups (n = 6): the vehicle control group (Veh), the CS exposure group (CS), CS-32 mg/kg swertiamarin group (32 swe) and CS-16 mg/kg swertiamarin group (16 swe). The doses of swetiamarin were applied according to previous study [10]. The exposure protocol was designed according to relevant report [24]. Briefly, the rats from CS, 32 swe and 16 swe groups were exposed to the smoke of one cigarette/rat once a day for 90 days. The rats from 32 and 16 swe groups were orally given 32 and 16 mg/kg/d swertiamarin for 90 days, respectively. Additionally, another 6 rats were orally given 32 mg/kg/d swertiamarin for 90 days but without CS treatment (swe control group) for evaluating the possible prostatic toxicities of sweriamarin. At the end of experimental period, the prostate tissue samples were collected, weighted and excised. One part fresh tissue was used for subsequent western blot and quantitative RT-PCR (qPCR) assay. One part tissue was fixed in 4% paraformaldehyde, embedded in paraffin and sectioned. One part tissue was made to the 10% tissue homogenate using ice cold 0.9% sodium chloride for the subsequent assay of prostatic inflammation and oxidative stress.

### Analysis for prostatic collagen deposition

The rat prostatic collagen deposition was assessed by prostate section sirius red staining, and by examining the prostate content of Hyp according to the manufacturer′s instructions. Additionally, the prostatic mRNA levels of Col1A1 and Col3A1 were detected by qPCR, and prostatic expression of Col-I and Col-III was measured by western blot. The total RNA was extracted from prostate samples using Trizol reagent (ThermoFisher Scientific) according to the manufacturer’s instructions. The qPCR was performed using a fast qPCR master mix kit in the real-time PCR system (ABI StepOne Plus). Post-PCR melt curve analysis was used to control the PCR product specificity. Relative levels were calculated based on the equation 2^-ΔΔCt^. The primer sequences of Col1A1 and Col3A1 were showed in Table 1. GAPDH was used as the internal standard.

**Table 1:** The primer sequences of α-SMA, E-cad, Col1A1 and Col3A1.

**gene**	**ID**	**forward primer (5′-3′)**	**reverse primer (5′-3′)**
α-SMA (Rattus norvegicus)	NM_031004.2	CGGGCATCCACGAAACCA	GAGCCGCCGATCCAGACA
E-cad (Rattus norvegicus)	NM_031334.1	TCACAGTCAAACGGCATCTAAA	5GGGCAGTTGATGGGAGGG
Col1A1 (Rattus norvegicus)	NM_053304.1	CCAGCGGTGGTTATG	CAGGCTCTTGAGGGTAG
Col3A1 (Rattus norvegicus)	NM_032085.1	GCCTCCCAGAACATTAC	CTTGCTCCATTCACCAG
GAPDH (Rattus norvegicus)	NM_017008.4	CAAGTTCAACGGCACAG	CCAGTAGACTCCACGACAT
α-SMA (Homo sapiens)	NM_001100.3	CGTGGCTACTCCTTCGTG	CGTCGCCATCTCGTTCT
E-cad (Homo sapiens)	NM_001317184.1	ACGCATTGCCACATACAC	ACCTTCCATGACAGACCC
Col1A1 (Homo sapiens)	NM_000088.3	CGAAGACATCCCACCAATC	ATCACGTCATCGCACAACA
Col3A1 (Homo sapiens)	NM_000090.3	CCCGTATTATGGAGATGAAC	TCAGGACTAATGAGGCTTTCT
GAPDH (Homo sapiens)	NM_001256799.3	CCACTCCTCCACCTTTG	CACCACCCTGTTGCTGT

The total protein were extracted from prostate samples and separated by 10% SDS-polyacrylamide gel electrophoresis. Then, the protein were transferred to a PVDF membrane *via* electrophoretic transferring and blocked for 1 h using 5% nonfat milk in Tris-buffered saline with 0.1% Tween 20 (TBST). Subsequently, the membranes were incubated with the primary antibody (Col-I or Col-III) overnight at 4 °C, washed with TBST and incubated with horseradish peroxidase-conjugated secondary antibodies in TBST with 3% nonfat milk for 0.5 h. The chemiluminescence reaction was developed and the quantification of bands was determined by integrated optical density analysis using Alpha Innotech software. The data were normalized using GAPDH as an internal control.

### Analysis for prostatic inflammation and oxidative stress

The prostatic oxidative stress was estimated by detecting the levels of the overall antioxidant status (TAOC and T-SH), the activities of antioxidant enzymes (SOD, CAT and GPx), the level of non-enzymatic antioxidant (GSH) as well as the contents of oxidative stress indicators (MDA and GSSG). The prostate local inflammation was evaluated by measuring the levels of proinflammatory cytokines (IL-1β, IL-6 and TNF-α) as well as the levels of inflammatory-related factors (COX-2, iNOS and NO). All the procedures were performed according to the manufacturer’s instructions.

### Analysis for prostatic EMT and HH signaling pathway

The prostatic EMT was analyzed by measuring the mRNA levels of EMT markers α-SMA and E-cad *via* qPCR and by detecting the protein expression of α-SMA and E-cad *via* western blot. The primer sequences of α-SMA and E-cad were showed in Table 1. GAPDH was used as the internal standard.

The activity of prostatic HH signaling pathway was analyzed by measuring the expression of HH signaling indicators (SHH, IHH, SMO and GLI-1), as well as its major target genes (EMT transcription factors Snail and ZEB1, promote-fibrosis cytokine TGF-β1 and extracellular matrix component Col-I) *via* western blot. GAPDH was used as the internal standard.

### Human prostate cells culture and CS exposure

WPMY-1 cells were cultured in DMEM with 5% FBS, 100 U/mL penicillin and 100 μg/mL streptomycin. RWPE-1 cells were maintained in keratinocyte serum-free medium containing 5 ng/mL epidermal growth factor and 0.01% gentamycin [25]. According to reported method, 10% CS extract was prepared by bubbling smoke from one cigarette into 10 mL medium and filtering from 0.22 μm microporous membrane [26].

Firstly, the cells were cultured with swertiamarin (1.5, 3, 6, 15, 30, 50 or 100 μmol/L) for 48 h and detected the survival rate by CCK8 method. Secondly, the cells were co-incubated with 5% CS extract and swertiamarin (1.5, 3, 6, 15 or 30 μmol/L) for 48 h. Then, the cells were collected and detected the survival rate by CCK8 method. Lastly, the cells were co-incubated with 5% CS extract and swertiamarin (15 or 30 μmol/L) or GDC-0449 (5 and 10 μmol/L) for 48 h. Then, the cells were collected for analyzing the expression of α-SMA, E-cad, Col-I, Col-III, SHH, IHH, SMO, GLI-1, Snail, TGF-β1 and ZEB1 by western blot, as well as for detecting the mRNA levels of α-SMA, E-cad, Col1A1 and Col3A1 by qPCR. The primer sequences of α-SMA, E-cad, Col1A1 and Col3A1 were showed in Table 1. GAPDH was used as the internal standard.

### Statistical analysis

The values were presented as mean ± S.D. Results were analyzed statistically by one-way ANOVA compare means using SPSS 17. Differences were considered as significant at *P*<0.05.

## Supplementary Material

Supplementary Figures
