# Supplementary material for: The anti-inflammation, anti-oxidative and anti-fibrosis properties of swertiamarin in cigarette smoke exposure-induced prostate dysfunction in rats
Source: Aging (Albany NY). 2019 Nov 17;11(22):10409–21. doi: 10.18632/aging.102467 (PMC6914396; doi:10.18632/aging.102467)
Supplement: Supplementary Figures [file aging-11-102467-s001..pdf]

SUPPLEMENTARY FIGURES

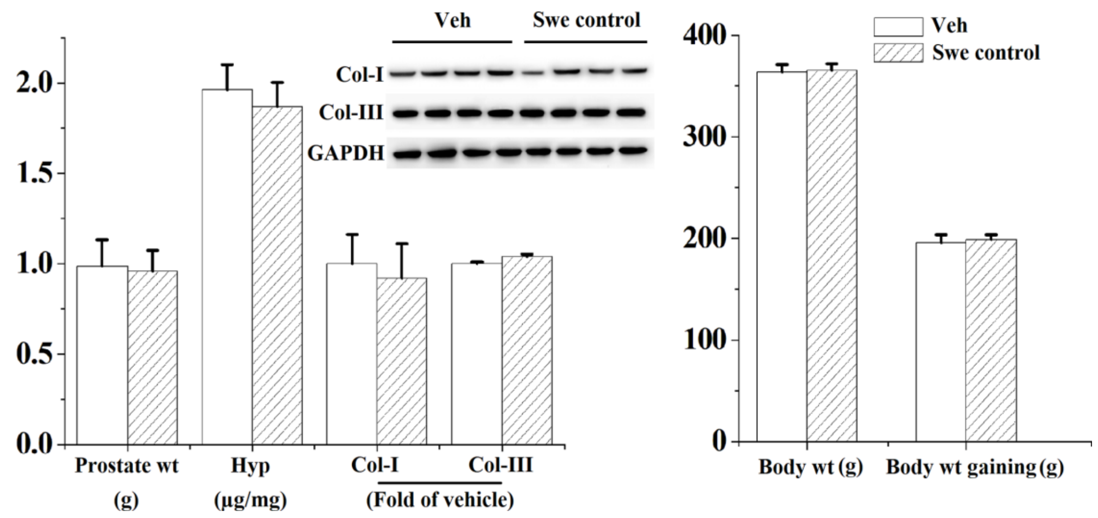

Supplementary Figure 1. Effects of sweriamarin on (n=6 per group) body weight (wt), body wt gaining, prostate wt and Hyp content, as well as on (n=4 per group) prostatic expression of Col-I and Col-III. Veh: vehicle, Swe Control: 32 mg/kg/d sweriamarin but without CS treatment.

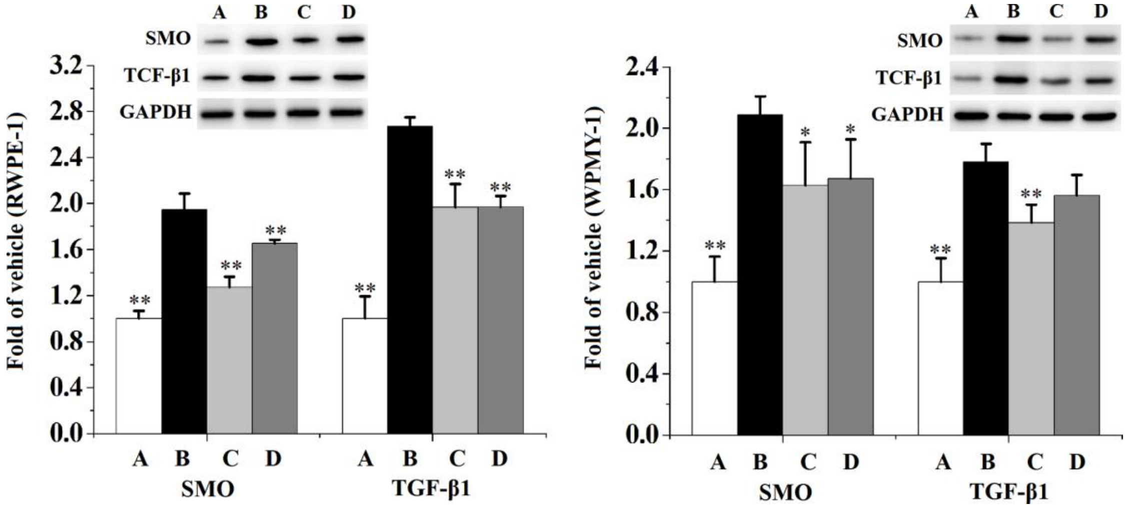

Supplementary Figure 2. Effects of sweriamarin on (n=4 per group) the expression of SHH and IHH in RWPE-1 and WPMY-1 cells. Veh: vehicle, Swe control: 30 μmol/L sweriamarin but without CS treatment.

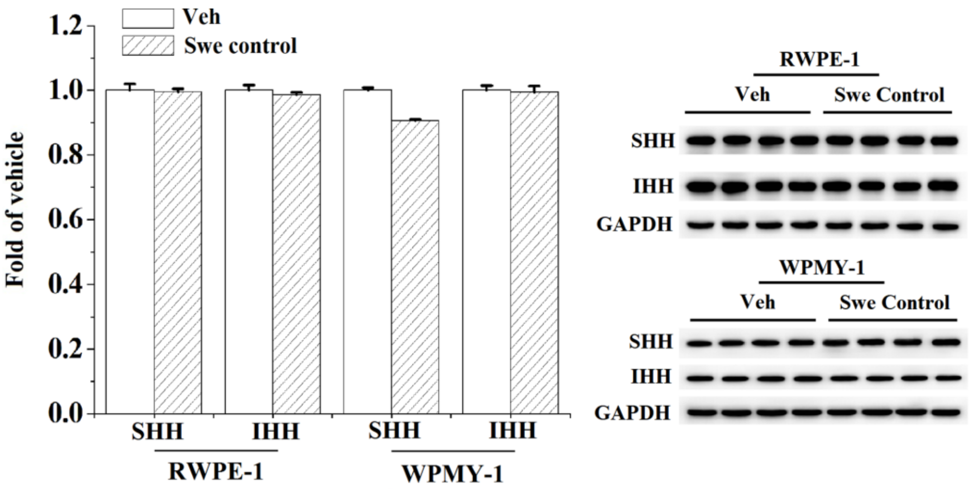

Supplementary Figure 3. GDC-0449 inhibited the (n=3 per group) expression of SMO and TGF-β1 in RWPE-1 and WPMY-1 cells. ** p<0.01 compared to the CS model group. * p<0.05 compared to the CS model group. A: vehicle, B: 5% CS, C: 5% CS-10 μmol/L GDC-0449, D: 5% CS-5 μmol/L GDC-0449.
